# Differential cytokine network profile in polycythemia vera and secondary polycythemia

**DOI:** 10.1038/s41598-020-63680-7

**Published:** 2020-04-27

**Authors:** Maira da Costa Cacemiro, Juçara Gastaldi Cominal, Maria Gabriela Berzoti-Coelho, Raquel Tognon, Natalia de Souza Nunes, Belinda Simões, Ítalo Sousa Pereira, Daniela Carlos, Lucia Helena Faccioli, Lorena Lobo de Figueiredo-Pontes, Fabiani Gai Frantz, Fabíola Attié de Castro

**Affiliations:** 1 Department of Clinical Analyses, Toxicology and Food Sciences, School of Pharmaceutical Sciences of Ribeirão Preto, University of São Paulo - USP, Ribeirão Preto - SP, Brazil; 2 Department of Pharmacy, Federal University of Juiz de Fora, Campus Governador Valadares, Governador Valadares - MG, Brazil; 3 Department of Basic and Applied Immunology, Ribeirão Preto Medical School, University of São Paulo - USP, Ribeirão Preto - SP, Brazil; 4 Department of Medical Images, Hematology and Oncology, Ribeirão Preto Medical School, University of São Paulo - USP, Ribeirão Preto - SP, Brazil

**Keywords:** Myeloproliferative disease, Interleukins

## Abstract

Polycythemia vera (PV) is a clonal disorder resulting from neoplastic transformation of hematopoietic stem cells, while secondary polycythemia (SP) is a disease characterized by increased absolute red blood cell mass caused by stimulation of red blood cell production. Although the physiopathology of SP and PV is distinct, patients with these diseases share similar symptoms. The early differential diagnosis may improve the quality of life and decrease the disease burden in PV patients, as well as enable curative treatment for SP patients. PV is considered an oncoinflammatory disease because PV patients exhibit augmented levels of several pro-inflammatory cytokines. In this sense, we examined whether analysis of the cytokine production profile of SP and PV patients would help to distinguish them, despite their clinical similarities. Here we reported that SP patients exhibited decreased plasma levels of, IL-17A, IFN-γ, IL-12p70 and TNF-α when compared with PV patients, suggesting that analysis of the cytokine production profile may be an useful diagnostic biomarker to distinguish PV from SP patients.

## Introduction

Polycythemia vera (PV) and secondary polycythemia (SP) are hematological diseases characterized by erythropoiesis exacerbation. PV patients present clonal expansion of the hematopoietic erythroid progenitor and, to a lesser extent, leukocytosis and/or thrombocytosis^1^. PV affects more women than men, has an incidence of 0.68–2.6 for every 100,000 individuals per year, and is uncommon in patients younger than 60 years. PV patients have life expectancy of 14 years after diagnosis, and their major risk factors for mortality are thrombotic events and leukemic transformation^2,3^. The augmented levels of pro-inflammatory cytokines in PV patients give this neoplasm the status of oncoinflammatory disease^4–6^.

Secondary polycythemia (SP) is a poorly understood clinical entity. The SP prevalence is considerably high, but it is hard to quantify due to the variety of causes and scarcity of data^7^. Compared with PV patients, SP patients have lower overall survival^8,9^ and higher morbidity and mortality rates, as demonstrated by small non-randomized studies. In spite of the epidemiological evidence, it is not clear whether the high mortality rate is associated with the increased red blood cell volume. To date, no randomized studies were conducted to analyze whether the morbidity and/or mortality rates in SP patients correlate with the increased red blood cell volume and thrombosis^7^.

SP can be congenital, caused by erythropoietin (EPO) receptor mutations, or acquired and induced by physiological changes that raise the body demand for oxygen, such as renal, lung and heart disease, high altitudes, severe obesity, defective oxygen transport, and EPO overproduction caused by certain kidney diseases or secretion by some tumors^8–10^. The increment in erythrocyte mass in SP patients results from bone marrow stimulation by EPO or abnormal functioning of the mutant EPO receptor. In contrast to PV patients, SP patients do not have increased leukometry, platelet levels or splenomegaly. SP is associated with cardiopulmonary disorders, chronic obstructive pulmonary disease, sleep apnea syndrome, smoker polycythemia, renal polycythemia of EPO-producing tumors, polycystic kidney disease, altitude polycythemia, and large fibroids.

Complementary exams, such as arterial blood gas analysis, abdominal ultrasound, sleep profile, and gynecological evaluation may help to perform the differential diagnosis between PV and SP^11^. In both diseases, erythrocytosis may cause blood hyperviscosity, which is associated with some disease symptoms like blurred vision, myalgia, weakness, fatigue, headache, and slowness of mental activity, as well as with the increased risk for thromboembolic and clotting events^10^. In this sense, the differential and early diagnosis of PV and SP is crucial.

The present study describes SP as a non-inflammatory disease and compares the differences between the cytokine profile in SP and PV patients.

### Ethical aspects

The Research Ethics Committee from the School of Pharmaceutical Sciences of Ribeirão Preto, University of São Paulo, Brazil, approved the study protocol (recorded under number CAAE 30901714.3.3001.5440), which complied with the guidelines established by the Brazilian National Health Council. All the patients and healthy individuals (controls) signed a written informed consent form to participate in this study.

### Subjects and blood collection

All healthy individuals are from Ribeirão Preto community and their demographic data are reported in Table S1. All the patients enrolled in this study are from Ribeirão Preto Medical School Hospital (HC-FMRP-USP), University of São Paulo, Ribeirão Preto, SP, Brazil, and their demographic, clinical hematological characteristics are reported in Table S2. The patients were divided into two groups according to the assay used to quantify plasma cytokine levels, as described below.

First group, 18 PV patients at diagnosis and 4 SP patients were enrolled to quantify cytokines using the multiplex platform. The PV group was composed of 7 men and 11 women with mean age of 65 years, who were positive for JAK2 mutation in exon 14 or exon 12; the PV patients with inherited causes of disease were excluded. The SP group was composed of two men and two women with mean age of 49 years, who were negative for JAK2 mutation in exon 14 or exon 12. The four SP patients developed the disease due to sleep apnea (2 patients), neuroma (1 patient), osteoarthritis (1 patient).

Second group, 16 PV patients at diagnosis and seven SP patients were enrolled to quantify cytokines using the ELISA assay. The PV group was composed of 6 men and 10 women with mean age of 67 years, who were positive for JAK2 mutation in exon 14 or exon 12; the patients with polycythemia vera related to inherited causes of disease were excluded. The SP group was composed of 4 men and 3 women with mean age of 56 years, who were negative for JAK2 mutation in exon 14 or exon 12. The 7 SP patients developed the disease due to sleep apnea (3 patients), neuroma (1 patient), osteoarthritis (1 patient), treatment of iron deficiency anemia (1 patient), and the use of androgens (1 patient).

Twenty milliliters of peripheral blood samples from all the control (CTRL) subjects, PV and SP patients were collected using EDTA tubes (Vacutainer^®^; Becton, Dickinson and Company). Plasma samples were obtained after total blood centrifugation at 400 × g for 10 min, at 4 °C (Eppendorf 5810 R Centrifuge, Germany), and stored at −80 °C until quantification of cytokines.

### Quantification of cytokines/chemokines using the multiplex platform

The cytokine/chemokine levels from 18 PV patients at diagnosis and 4 SP patients were determined using the multiplex assay. A customized multiplex assay kit was used to determine the plasma concentration of granulocyte-macrophage colony-stimulating factor (GM-CSF), interleukin (IL) 5 (IL-5), IL-12p70, IL-17A, interferon (IFN) α2 (IFN-α2), IFN-γ, IFN-γ-induced protein 10 (IP-10), monocyte chemoattractant protein-1 (MCP-1), macrophage inflammatory protein (MIP) 1α (MIP-1α) and MIP-1β, RANTES (*regulated upon activation, normal T cell expressed and presumably secreted*), and tumor necrosis factor alpha (TNF-α) (12-plex, EMD Millipore Corporation, Massachusetts, USA). All the reagents used were part of the assay kit. Briefly, 200 µL/well of wash buffer was added to the 96-well plate, which was shaken for 10 min, at room temperature. The content was removed and 25 µL of standards and controls were added to the respective wells, followed by 25 µL of serum matrix; or 25 µL of assay buffer were added to the assay wells, followed by 25 µL of patients’ plasma. Next, 25 µL of beads were added to all the wells and the plate was incubated overnight at 4 °C, under shaking. The content was removed, and the plate was washed twice with 200 µL/well of wash buffer, before adding 25 µL/well of detection antibodies. After 1 h of incubation at room temperature, 25 µL/well of streptavidin-phycoerythrin were added and the plate was incubated for further 30 min, at room temperature. The content was removed, the plate was washed twice with 200 µL/well of wash buffer, and 150 µL/well of sheat fluid were added. Fluorescence was recorded using the Luminex MAGPIX1 System equipment (Luminex Corporation, Texas, USA), and data were analyzed using the Milliplex Analyst software v3.5 (Millipore; Vigene Tech Inc., Boston, Massachusetts, USA).

### Quantification of cytokines/chemokines using ELISA assay

To validate the findings obtained by cytokines/chemokines dosage using the multiplex platform, we performed ELISA assay for the most secreted (plasmatic) cytokines in PV. The IFN-γ, IL-12p70, IL-17A, and TNF-α levels from 16 PV patients at diagnosis and seven SP patients were determined using the enzyme-linked immunosorbent assay (ELISA), in order to validate the cytokine/chemokine patterns found in PV and SP patients through the multiplex assay. One 96-well plate was used to quantify each cytokine.

First, the wells were coated with the specific capture antibody for the cytokine of interest. The plate was incubated overnight and washed twice with phosphate-buffered saline (PBS)/Tween solution and once with PBS. Standard cytokine samples, control samples, and plasma samples were added to the wells and the plate was incubated for 1 h to allow the antigen binding to the capture antibody. The plate was washed twice with PBS/Tween and once with PBS before adding the detection antibody and incubated for 2 h to allow its binding to the immobilized antigen captured during the first incubation period. The plate was washed twice with PBS/Tween and once with PBS. HRP conjugated with streptavidin was added to the wells and incubated for 40 minutes to allow its binding to the detection antibody. After washing twice with PBS/Tween and once with PBS to remove the excess of HRP conjugate, a substrate solution was added to the wells and converted into a detectable product (color signal). Absorbance was recorded in the SpectraMax^®^ Paradigm^®^ Multi-Mode Microplate Detection Platform (Molecular Devices), at 450 nm. The intensity of the colored product is directly proportional to the antigen concentration in the original sample.

### Data analyses

The Mann-Whitney test was used to compare the levels of plasma cytokines and gene expression in PV and SP patients. Statistical analyses were carried out using the GraphPad Prism 8.0 software (Graph-Pad Software, San Diego, CA, USA) and the differences were considered significant when p ≤ 0.05.

The cytokine profile was analysed to characterize the overall pattern of cytokine production in each group, as suggested by Vitelli-Avelar *et al*. (2008). These authors grouped the cytokine/chemokine production percentages using a four-step platform, which we modified as follows: (I) conventional statistical analysis; (II) establishment of the global median cytokine/chemokine levels for each group; (III) classification of each group as low or high cytokine/chemokine producers, using the global median value as the cutoff value; (IV) creation of black and white scale diagrams for each group. These approaches are relevant to detect, with high sensitivity, small changes in immunological profiles that are not detectable by conventional statistical approaches^12–14^. The mean concentration value for each cytokine/chemokine was calculated and used as the cutoff value to classify each individual (patient or control) as “high” or “low” producer of a given cytokine/chemokine. Then, after classifying each patient/control as high or low producer of a specific cytokine/chemokine, we analyzed how relevant it was for the disease pathology. To consider a given cytokine/chemokine as relevant, ≥50% of patients (≥50% frequency) with a given disease (e.g. PV and SP) must be high producers of it. Relevant data (≥50% frequency) were highlighted in bold and underlined in the figures. Radar charts were plotted using Microsoft Excel (Microsoft Office 2016) to characterize the overall frequency of individuals (controls and patients) who expressed high levels of each cytokine/chemokine in each group studied.

## Results

### PV patients exhibit inflammatory profile when compared with healthy subjects and SP patients

**Figure 1:**
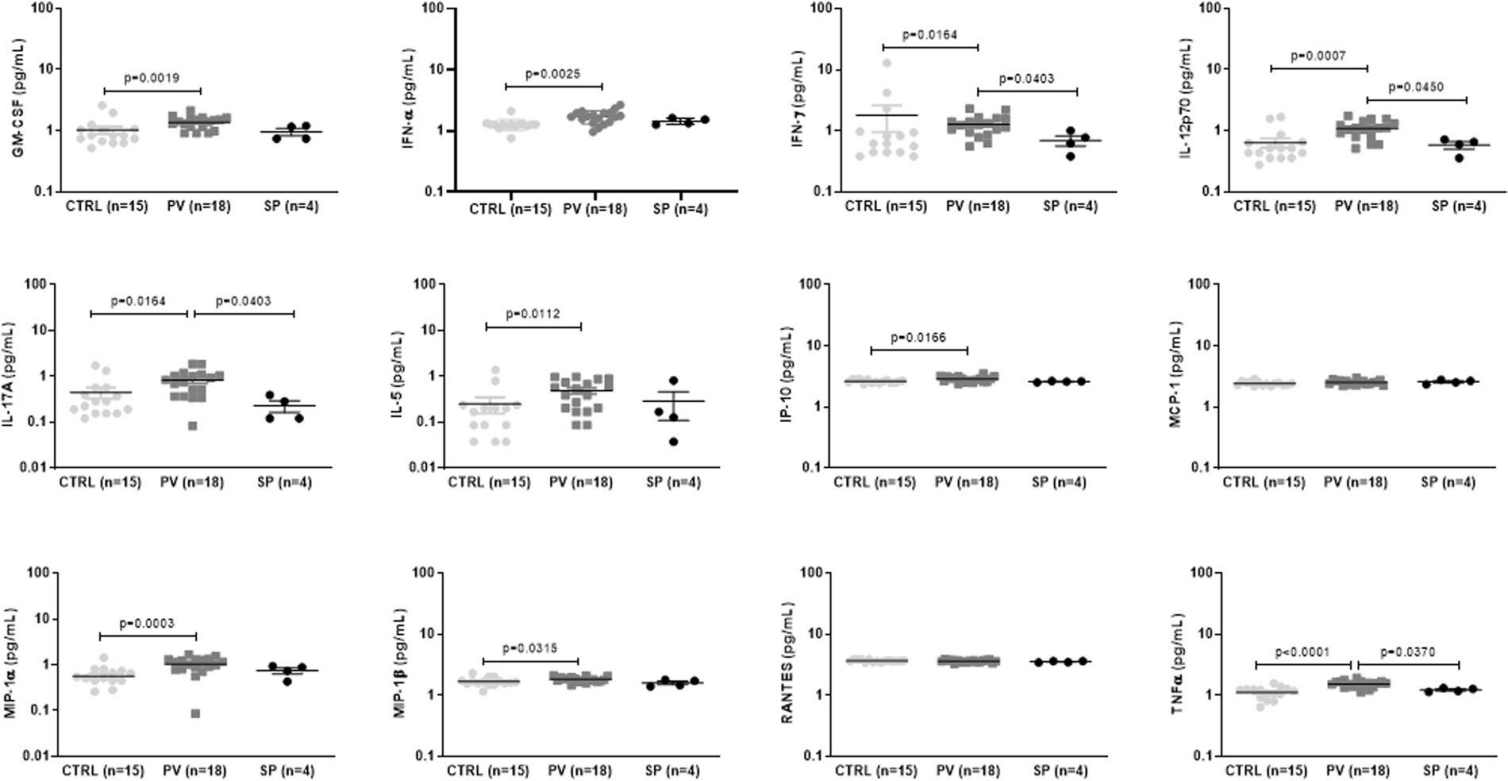
Plasma cytokine levels in patients with polycythemia vera (PV) and secondary polycythemia (SP), and in healthy subjects (CTRL). SP patients exhibited lower levels of IL-17A, IFN-γ, IL-12p70, and TNF-α than PV patients.

Compared with the control group, PV patients exhibited increased levels of GM-CSF (p = 0.0019), IFN-α2 (p = 0.0005), IFN-γ (p = 0.0164), IL-12p70 (p = 0.0007), IL-17A (p = 0.0164), IL-5 (p = 0.0112), IP-10 (p = 0.0166), MIP-1α (p = 0.0003), MIP-1β (p = 0.00315) and TNF-α (p < 0.0001) (Fig. 1). Compared with SP patients, PV patients displayed higher levels of IFN-γ (p = 0.0403), IL-12p70 (p = 0.0450), IL-17A (p = 0.0403) and TNF-α (p = 0.0370) (Fig. 1). The mean concentration of these cytokines/chemokines and their range in each group are reported in Table S3.

### Differential cytokine patterns in PV and SP patients

**Figure 2:**
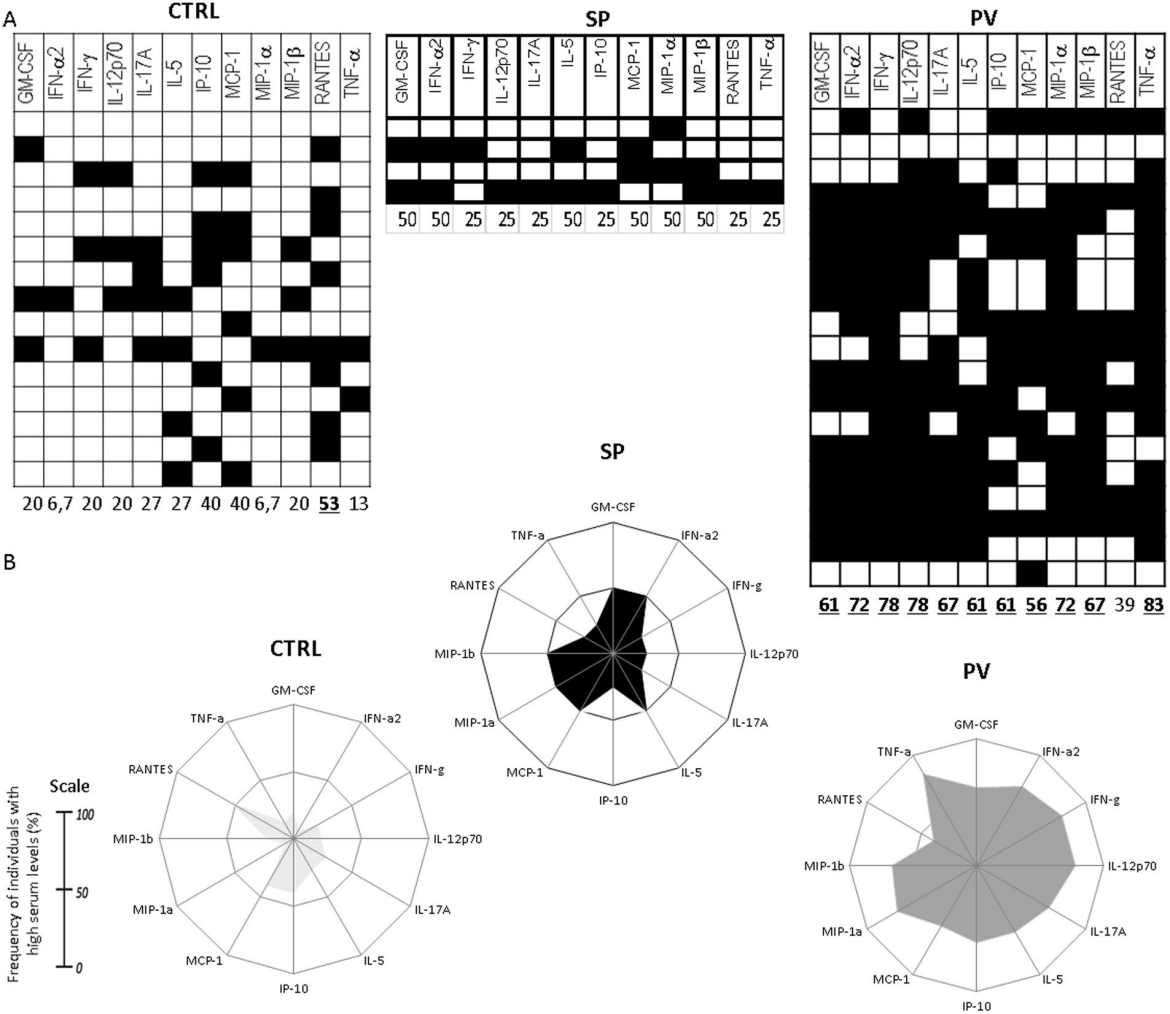
Cytokine profile in patients with polycythemia vera (PV) and secondary polycythemia (SP). (**A**) Black-and-white diagram of the plasma levels of GM-CSF, IFN-α, IFN-γ, IL-5, IL-12p70, IL-17A, IP-10, MCP-1, MIP-1α, MIP-1β, RANTES, and TNF-α used for categorical classification of PV and SP patients as high and low producers of a given cytokine. Each column represents a cytokine, each row represents the cytokine production pattern of one patient, and the black and white blocks represent high and low producers of each cytokine, respectively. The numbers at the bottom of each column represent the frequency of high producers of the cytokine tested. (**B**) Radar charts represent the immune profile of patients with PV and SP and summarize the percentage of high producers of each cytokine in the two clinical groups studied.

To better characterize the immune profiles of PV, SP and control groups, their respective data were plotted in black-and-white scale diagrams and radar charts (Fig. 2). Except for RANTES, all the other cytokines analyzed seemed to play important roles in the PV disease process because more than 50% of the PV patients were considered as high cytokine producers. In contrast, the SP patients’ group was composed of low producers of all the cytokines quantified.

### The differential cytokine patterns in PV and SP patients were validated by ELISA assay

To confirm the differential cytokine patterns in PV and SP patients, the levels of IFN-γ, IL-12p70, IL-17A, and TNF-α were quantified using the ELISA assay. Compared with SP patients, PV patients displayed increased levels of IFN-γ (p = 0.0021), IL-12p70 (p = 0.0212), and TNF-α (p = 0.0311) (Fig. 1S), in agreement with the findings reported in Fig. 2. The IL-17A presented similar levels in plasma from PV and SP patients. It is important to note that the differential IL-17A expression between PV and SP patients was detected only using the multiplex platform, which reinforces that this method is more sensible than the ELISA assay. The mean concentration of each cytokine analyzed, and their range are reported in Table S4.

## Discussion

The findings from the present investigation reinforce that PV patients exhibit a pro-inflammatory profile when compared with healthy individuals, as previously reported by other authors^4,15,16^. In addition, this study demonstrated the differential cytokine profiles in PV and SP patients: compared with the former, the latter had a non-inflammatory profile characterized by decreased levels of IL-17A, IFN-γ, IL-12p70, and TNF-α.

It is worthy to emphasize that the IL-17A higher levels is only detected in PV patients when we used the multiplex platform. We did not observe the same result using ELISA methodology. This data reinforces that multiplex platform method is more sensible than the ELISA assay.

IL-17A plays a critical role in the host defense against various microbial pathogens, tissue inflammation, autoimmunity^17^, and the regulation of hematopoietic stem cell fate^18^. The combined effect of IL-17 and TNF-α contributes to amplify pro-inflammatory responses through stimulation of IL-6 and IL-8 secretion^19^. A previous study from our research group reported the augmented levels of IL-17A in primary myelofibrosis patients, as compared with essential thrombocythemia patients and healthy subjects^4^. However, there are few reports on the role that IL-17A plays in hematological diseases. IL-17A may be beneficial in chronic lymphocytic leukemia development, since its levels are inversely correlated with the time between the disease diagnosis and start of the therapy, and its levels are lower in patients who require treatment during the follow-up^20^.

IFN-γ is a cytokine produced mainly by activated CD4^+^ or CD8^+^ T cells and natural killer cells. IFN-γ participates in both innate and adaptive immunity and enhances activation of the pro-inflammatory transcription of nuclear factor κB^21^. The IFN-γ production is associated with thrombocytosis in primary myelofibrosis patients^5^. In acute myeloid leukemia cells, IFN-γ affects cell proliferation, apoptosis regulation, and the balance between pro- and antiangiogenic chemokine release^22^. The IFN-γ gene expression is lowered in patients with acute lymphoblastic leukemia, suggesting an immune system disruption that may favor evasion of tumor cells from immune surveillance^23^.

IL-12 is produced by monocytes, macrophages, dendritic cells, and B cells. IL-12 elicits IFN production by natural killer and T cells and strengthens their cytotoxic effects, as well as induces the differentiation of naïve T cells into Th1 effectors^24^. Predisposition to thrombotic events is a common feature of myeloproliferative neoplasms^25^. Low IL-12p70 levels are associated with increased risk for vascular complications in PV patients, indicating that this cytokine protects against thrombotic events^16,26^.

TNF-α is mainly produced by monocytes and macrophages and it is not usually detectable in healthy individuals, in fact, it is associated with severe infections, when high serum and tissue levels are detected^27^. PV patients bearing the JAK2V617F mutation exhibit higher TNF-α levels than patients not bearing this mutation^16^. TNF-α promotes clonal predominance of JAK2V617F-expressing cells from myeloproliferative neoplasms^28^. This cytokine stimulates proliferation of the JAK2V617F-positive primary progenitor cells and inhibits colony formation in normal control cells, as assessed by clonogenic assays, as well as it enhances the expansion of JAK2V617F cells in a murine model of myeloproliferative neoplasm^28^.

The GM-CSF levels in PV patients are higher than the normal physiological levels of human cytokines measured under the same conditions^16^. In addition, the GM-CSF levels are decreased in PV patients with vascular complications, which probably reflects a cellular microenvironment dysfunction that impairs the hemostatic balance^16^.

Only PV patients bearing the JAK2 mutation were selected for the present study. The presence of the JAK2V617F mutation may influence cytokine production in patients with myeloproliferative neoplasms^4,6,19^. This mutation correlates with elevated levels of IL-1RA, IL-2R, IL-6, IL-12, IP-10, hepatocyte growth factor, and monokine induced by IFN-γ in primary myelofibrosis patients^6^. Compared with non-mutated patients, PV and patients bearing the JAK2V617F mutation display lower TNF-α levels and higher platelet-derived growth factor-BB levels^19^. A previous study from our research group has reported the elevated IP-10 level in JAK2V617F-positive primary myelofibrosis patients, as compared with JA2V617F-negative cells^4^.

Altogether, the findings reported herein demonstrated that the levels of IL-17A, IFN-γ, IL-12p70, and TNF- α were increased in PV patients, which reinforces the oncoinflammatory characteristic of PV and suggests that such inflammatory status is a useful marker to help physicians to distinguish PV patients from SP patients. In fact, we do not suggest quantification of the aforementioned cytokines to diagnose PV but, considering that PV is an oncoinflammatory disease, we propose their quantification as complementary laboratory exam to help physicians to distinguish PV from SP patients.

### Ethics approval and consent to participate

We declare that The Human Research Ethics Committee of the School of Pharmaceutical Sciences of Ribeirão Preto, University of São Paulo, Brazil, approved this study protocol, which was registered under the code CAAE 30901714.3.3001.5440. We declare that the study was conducted with the consent of the human participants.

## Conclusion

The increased plasma levels of the cytokines IL-17A, IFN-γ, IL-12p70, and TNF- α are potential biomarkers of oncoinflammation in PV patients that can be useful to distinguish them from SP patients.

## Supplementary information

Differential cytokine network profile in polycythemia vera and secondary polycythemia.

## Data Availability

All data generated or analyzed during this study are included in this published article.
