# Supplementary material for: Differential cytokine network profile in polycythemia vera and secondary polycythemia
Source: Sci Rep. 2020 Apr 27;10:7032. doi: 10.1038/s41598-020-63680-7 (PMC7468352; doi:10.1038/s41598-020-63680-7)
Supplement: Supplementary file 1 — Differential cytokine network profile in polycythemia vera and secondary polycythemia. [file 41598_2020_63680_MOESM1_ESM.docx]

**SUPPLEMENTARY MATERIAL**

**TITLE PAGE**

**Title:** **Differential cytokine network profile in polycythemia vera and secondary polycythemia**

**Short title**: **Cytokine profile in secondary polycythemia**

Authors:

Maira da Costa Cacemiro^1^* (mairacacemiro@hotmail.com)

Juçara Gastaldi Cominal^1^ (cominal@fcfrp.usp.br)

Maria Gabriela Berzoti-Coelho^1^ (gabrielaberzoti@usp.br)

Raquel Tognon^2^ (raqueltognon@hotmail.com)

Natalia de Souza Nunes^1^ (natisnunes@gmail.com)

Belinda Simões^4^ (bpsimoes@fmrp.usp.br)

Ítalo Sousa Pereira^3^ (italo.biotecnologista@gmail.com)

Daniela Carlos ^3^ (danicar@usp.br)

Lucia Helena Faccioli^1^ (faccioli@fcfrp.usp.br)

Lorena Lobo de Figueiredo-Pontes^4^ (lorenafgdo@yahoo.com.br)

Fabiani Gai Frantz^1^ (frantz@fcfrp.usp.br)

Fabíola Attié de Castro^1^* (castrofa@fcfrp.usp.br)

^1^Department of Clinical Analyses, Toxicology and Food Sciences, School of Pharmaceutical Sciences of Ribeirão Preto, University of São Paulo - USP, Ribeirão Preto - SP, Brazil

^2^Department of Pharmacy, Federal University of Juiz de Fora, Campus Governador Valadares, Governador Valadares - MG, Brazil.

^3^Department of Basic and Applied Immunology, Ribeirão Preto Medical School, University of São Paulo - USP, Ribeirão Preto - SP, Brazil

^4^Department of Internal Medicine, Ribeirão Preto Medical School, University of São Paulo - USP, Ribeirão Preto - SP, Brazil

*Corresponding Authors: Maira da Costa Cacemiro, Ph.D, and Fabíola Attié de Castro, PhD

Laboratório de Fisiopatologia das Doenças Hematológicas

Departamento de Análises Clínicas, Toxicológicas e Bromatológicas. Faculdade de Ciências Farmacêuticas de Ribeirão Preto, Universidade de São Paulo - USP.

Av. do Café, s/nº. Campus Universitário, Ribeirão Preto – SP, 14040-903, Brasil.

Telephone number: +55 (16) 3315-0657

Fax number: +55 (16) 3315-4725

E-mail: mairacacemiro@hotmail.com (MC Cacemiro) and castrofa@fcfrp.usp.br (FA Castro)

**Supplementary Material**

Table S1. Demographic data of the controls studied.

| **Patient** | **Age (years)** | **Gender** | **Mutation status** |
| --- | --- | --- | --- |
| CTRL 1 | 50 | Male | Negative |
| CTRL 2 | 49 | Female | Negative |
| CTRL 3 | 83 | Female | Negative |
| CTRL 4 | 31 | Female | Negative |
| CTRL 5 | 66 | Female | Negative |
| CTRL 6 | 58 | Male | Negative |
| CTRL 7 | 54 | Female | Negative |
| CTRL 8 | 72 | Male | Negative |
| CTRL 9 | 38 | Female | Negative |
| CTRL 10 | 60 | Female | Negative |
| CTRL 11 | 56 | Female | Negative |
| CTRL 12 | 61 | Female | Negative |
| CTRL 13 | 65 | Male | Negative |
| CTRL 14 | 60 | Female | Negative |
| CTRL 15 | 52 | Male | Negative |

Table S2. Demographic and clinical hematological parameters of the patients with polycythemia vera (PV) and secondary polycythemia (SP) studied.

| **Patient** | **Age** | **Gender** | **Mutation status** | **Disease type** | **WBC (10^3^/µL)** | **RBC**  **(10^6^/µL)** | **Hb (g/dL)** | **Ht**  **(%)** | **Plt**  **(10^3^/µL)** |
| --- | --- | --- | --- | --- | --- | --- | --- | --- | --- |
| PV 1 | 54 | Female | JAK2V617F | PV | 7.8 | 6.5 | 16.4 | 49.6 | 288 |
| PV 2 | 60 | Female | JAK2V617F | PV | 9.8 | 5.2 | 16.9 | 50.7 | 440 |
| PV 3 | 83 | Female | JAK2V617F | PV | 21.3 | 7.3 | 16.0 | 53.9 | 420 |
| PV 4 | 76 | Male | JAK2V617F | PV | 8.9 | 5.7 | 16.6 | 49.1 | 181 |
| PV 5 | 75 | Male | JAK2V617F | PV | 6.8 | 3.6 | 11.3 | 33.7 | 483 |
| PV 6 | 69 | Male | JAK2V617F | PV | 18.4 | 7.1 | 11.1 | 42.7 | 605 |
| PV 7 | 57 | Male | JAK2V617F | PV | 3.6 | 7,09 | 17,5 | 56,7 | 664 |
| PV 8 | 51 | Female | JAK2V617F | PV | 6.4 | 5.5 | 15.5 | 47.0 | 449 |
| PV 9 | 79 | Male | JAK2V617F | PV | 15.7 | 7.4 | 19.5 | 60.4 | 772 |
| PV 10 | 61 | Male | JAK2V617F | PV | 9.7 | 6.6 | 21.5 | 62.0 | 161 |
| PV 11 | 65 | Female | JAK2V617F | PV | 7.3 | 4.9 | 15.5 | 48.5 | 531 |
| PV 12 | 69 | Female | JAK2V617F | PV | 14.2 | 4.1 | 13.2 | 40.1 | 621 |
| PV 13 | 61 | Female | JAK2V617F | PV | 6.4 | 4.6 | 15.5 | 48.2 | 451 |
| PV 14 | 78 | Female | JAK2V617F | PV | 15 | 6.6 | 13.5 | 46.5 | 314 |
| PV 15 | 67 | Male | JAK2V617F | PV | 21 | 6.4 | 17.7 | 55.9 | 591 |
| PV 16 | 72 | Female | JAK2V617F | PV | 5.5 | 6.6 | 9.9 | 30.0 | 327 |
| PV 17 | 57 | Female | JAK2V617F | PV | 17.5 | 6.41 | 17.5 | 51.0 | 575 |
| PV 18 | 49 | Female | JAK2V617F | PV | 10.8 | 5.52 | 17.1 | 51.0 | 254 |
| SP 1 | 39 | Female | Negative | SP | 9.8 | 4.3 | 14.3 | 41.6 | 461 |
| SP 2 | 47 | Male | Negative | SP | 3.6 | 5.8 | 18.1 | 50.7 | 211 |
| SP 3 | 45 | Male | Negative | SP | 7.5 | 5.8 | 16.3 | 48.0 | 267 |
| SP 4 | 68 | Female | Negative | SP | 9 | 6.7 | 16.8 | 49.0 | 577 |
| SP 5 | 41 | Male | Negative | SP | 6.3 | 7.0 | 21.3 | 62.0 | 213 |
| SP 6 | 56 | Male | Negative | SP | 8.4 | 5.3 | 17.2 | 50.0 | 264 |
| SP 7 | 80 | Female | Negative | SP | 15.3 | 4.54 | 15.7 | 47.0 | 95 |

PV: polychythemia vera; SP:secondary polycythemia; Hb: hemoglobin. Ht: hematocrit. Plt: platelets. RBC: red blood cells. WBC: white blood cells.

Table S3. Plasma concentration of cytokines/chemokines in patients with polycythemia vera (PV) and secondary polycythemia (SP) and control subjects (CTRL), as determined using multiplex analysis.

| **Cytokine/**  **Chemokine** | **Plasma concentration (pg/µL)** | | |
| --- | --- | --- | --- |
|  | **CTRL** | **PV** | **SP** |
| GM-CSF | 14.43 (3.29 - 390.07) | 22.61 (5.48 - 135.66) | 15.11 (5.48 - 30.77) |
| IFN-α2 | 21.64 (5.79 - 127.39) | 49.63 (9.17 - 459.33) | 38.34 (18.61 - 57.69) |
| IFN-γ | 8.64 (2.41 - 197.82) | 17.47 (2.41 - 197.82) | 6.60 (4.18 - 10.01) |
| IL-12p70 | 5.20 (1.88 - 47.18) | 11.58 (2.7 - 54.86 | 4.84 (3.92 - 12.94) |
| IL-17A | 2.46 (1.32 - 52.83) | 6.24 (1.21 - 74.19) | 2.19 (1.32 -3.24) |
| IL-5 | 1.60 (0.86 - 24.53) | 2.94 (1.09 - 9.17) | 1.95 (1.34 - 6.47) |
| IP-10 | 447.93 (224.32 - 695.55) | 759.79 (235.89 – 1720) | 421.46 (327.11 - 534.14) |
| MCP-1 | 286.03 (148.14 - 740.69) | 344.22 (155.78 – 1027) | 286.03 (218.75 - 590.67) |
| MIP-1α | 6.47 (0.87 - 27.47) | 12.55 (2.71 - 48.38) | 6.58 (3.62 - 8.64) |
| MIP-1β | 53.50 (14.43 - 202.85) | 60.03 (25.16 - 132.29) | 58.65 (29.26 - 67.09) |
| RANTES | 4284.00 (2767.00 – 7830.00) | 4222.50 (1950.00 – 8505.00) | 3740.50 (2931.00 – 5528.00) |
| TNF-α | 21.10 (4.37 - 37.62) | 35.19 (13.3 - 86.27) | 19.97 (13.67 - 34.02) |

CTRL: control; PV: polychythemia vera; SP:secondary polycythemia. Results are expressed as median and range (in parentheses).

Table S4. Plasma concentration of cytokines/chemokines in patients with polycythemia vera (PV) and secondary polycythemia (SP), as determined using the enzyme-linked immunosorbent assay (ELISA).

| **Cytokine/**  **Chemokine** | **Plasma concentration (pg/µL)** | |
| --- | --- | --- |
|  | **PV** | **SP** |
| IFN-γ | 8.47 (5.75 – 25.83) | 6.07 (5.41 – 7.15) |
| IL-12p70 | 10.29 (6.88 – 26.60) | 7.23 (6.65 – 8.04) |
| IL-17A | 10.31 (4.42 – 46.57) | 5.63 (5.24 – 6.54) |
| TNF-α | 6.29 (3.04 – 40.70) | 3.11 (2.28 – 3.54) |

PV: polychythemia vera; SP:secondary polycythemia. Results are expressed as median and range (in parentheses).

Figure S1. Comparative analysis of plasma concentration of cytokines/chemokines in patients with polycythemia vera (PV) and secondary polycythemia (SP), as determined using the ELISA assay. The cytokines IL12p70, IFN-α and TNF-α were more expressed in PV patients than in SP patients (p<0.05).
